# Needle knife therapy plus sodium hyaluronate injection for knee osteoarthritis: A protocol for a systematic review and meta-analysis

**DOI:** 10.1097/MD.0000000000023242

**Published:** 2020-11-13

**Authors:** Kunming Xie, Xuyue Pan, Fasen Huang, Yufeng Ma, Yinze Qi, Junde Wu, Zhanhua Ma, Xinyu Li, Huan Liang, Shulong Wang, Zhen Lei, Jingwei Tao, Hanjie Zhu, Zhaojun Chen

**Affiliations:** Third Affiliated Hospital of Beijing University of Chinese Medicine, Beijing, China.

**Keywords:** knee osteoarthritis, needle knife, protocol, sodium hyaluronate, systematic review

## Abstract

**Background:**

Knee osteoarthritis (KOA) is a worldwide disease and more and more people are suffered from it. With the increasing number of patients, it brings a huge burden on social economy and security system. There are varieties of methods to cure KOA, such as Traditional Chinese Medicine and surgery. Needle knife therapy plus Sodium hyaluronate Injection is one of the prevalent treatments for KOA. Therefore, we perform a systematic review and meta-analysis to evaluate the evidence for the treatment of needle knife therapy plus sodium hyaluronate Injection.

**Methods:**

Randomized controlled trials will be used to compare the effect of needle knife therapy plus sodium hyaluronate injection with needle knife alone for KOA patients. Six studies will be included in this meta-analysis, and the relative risk and weight mean difference with 95% CI for the Lysholm knee score, visual analogue scale, and effective rate will be evaluated by using RevMan 5.3 software. Besides, the bias assessment of the included studies will be evaluated using the Cochrane risk of bias tool, and the Grading of Recommendations, Assessment Development, and Evaluation system will be applied to assess the overall quality of the evidence.

**Results:**

From the study we will assess the effectiveness, safety of needle knife therapy plus sodium hyaluronate injection on joint pain relief and functional improvement in patients with KOA.

**Conclusion:**

The study will provide a new evidence to confirm the effect of needle knife therapy plus sodium hyaluronate injection on KOA, which can further guide the selection of therapy.

**PROSPERO registration number:**

CRD42020169602

## 1 Introduction

Knee osteoarthritis (KOA) is a kind of osteoarthropathy that often occurs in the elderly. Its main clinical manifestations are persistent swelling and dysfunction. It belongs to the category of “Bi syndrome” in traditional Chinese medicine (TCM), which is “bone Bi syndrome.”^[1]^ The etiology of KOA not only relates to age, gender, occupation, and education, but also physical fitness, heredity, excessive exercise, and other factors.^[2]^ With the aging of the population and the prolongation of the human beings’ life, the morbidity of osteoarthritis is not only high but also rising. The main risks of KOA are age, obesity, gender, and so on, which seriously affect the patients’ quality of life, causing depression and anxiety easily.^[3]^ The prevalence of KOA is about 12.4% to 15%.^[4,5]^ As the global population ages and obesity grows, KOA patients are expected to grow^[6]^ in the future. There are more than 27 million osteoarthritis patients in the United States, and it costs $185.5 billion every year, which brings a huge economic burden. The TCM believes that the etiology and pathogenesis of KOA is based on the deficiency of liver and kidney, deficiency of qi and blood, and invasion of wind, cold, and dampness, which should belong to the category of “bone arthralgia,” “gluten arthralgia,” and “pain arthralgia.” Due to the complexity of the pathological process, it is difficult to classify KOA and there is no consensus classification standard at present, so that dozens of types are proposed in recent. Generally speaking, KOA can be divided into 2 categories: primary and secondary. The primary pathogenic factor is usually considered to be related to gene phenotype or variation, but the concrete reason is still not clear.^[7]^ The primary KOA is classified into 3 categories: the genetically determined, estrogen hormone dependent, and aging related.^[8]^ The secondary is classified into 6 phenotype: the chronic pain type with the central mechanisms; the inflammatory type with high levels of inflammatory biomarker; metabolic syndrome type with diabetes, obesity, and hyperlipidemia; cartilage metabolism type with alteration in local tissue metabolism; mechanical overload type characterized primarily by varus malalignment and medial compartment disease; minimal joint disease type characterized as minor clinical symptoms with slow progression over time.^[9]^ In China, KOA is divided into 4 types in the diagnosis and treatment plan of knee arthralgia disease by China Administration of traditional Chinese medicine: wind cold dampness, rheumatism heat, liver kidney deficiency, and blood stasis.^[10]^ However, there are varieties of syndrome types that affect each other, and the situation of deficiency and excess are mixed in clinic.

The treatment methods of KOA for patients include TCM and Western medicine. Traditional Chinese medicine therapy includes oral Chinese medicine, fumigation and washing, external application, acupuncture and moxibustion, needle knife, and massage.^[11]^ The treatment of Western medicine includes oral medicine, extra-corporeal shock wave therapy, radio frequency therapy, surgical treatment, rehabilitation therapy, and functional exercise. Sodium hyaluronate (SH) is a kind of inherent high molecular polysaccharide of human body, which widely exists in dermis, lens, articular cartilage, and other tissues. It is one of the components of cartilage matrix that can be used to lubricate joints, protect articular cartilage, and improve joint spasm. In recent years, more and more researches have shown that SH is good at promoting tendon and bone healing, accelerating the formation of cartilage, increasing the expression of type I collagen, and promoting tendon maturation to enhance its biomechanical strength. Scar tissue and tendon bone were also rehabilitated by SH through reducing inflammation.^[12–14]^ Except for the function of anti-inflammatory, SH is able to inhibit the proliferation of fibroblasts, reduce the density of collagen matrix, slow down the progress of fibrosis, lubricate joints, improve mechanical and biological functions, avoid contracture and adhesion of joint capsule, and reduce the joint stress.^[15,16]^ Needle knife is a closed minimally invasive surgery, which integrates the acupuncture in modern Chinese medicine with the knife in surgery. It can restore the biomechanical balance, release the soft tissue, improve the internal microcirculation, reduce the internal pressure of bone, alleviate the inflammatory reaction, and regulate the meridians.^[17–19]^ Compared with traditional acupuncture and moxibustion, needle knife has absorbed and exerted the advantages of modern anatomy, bone injury biomechanics, pathology, aseptic theory, and anesthesia. People who suffer from musculoskeletal and connective tissue diseases can be better treated with needle knife.^[20]^ As a minimally invasive Chinese medicine, needle knife can effectively loosen the tendon, ligament, capsule, and other soft tissues.^[21]^ After soft tissue injury or pathological change, the adhesion and scar can be produced, which change the position and direction of ligament and fascia, destroy the static and dynamic balance, cause pain and dysfunction. The needle knife can peel off adhesion, release ligament, and fascia, restore local blood circulation, decrease bradykinin, 5-hydroxytryptamine, and then achieve a new static and dynamic balance.^[22]^

Needle knife therapy plus SH injection is also a common method for KOA treatment, but whether the combined application of these 2 methods is better than needle knife alone is still lack of systematic evaluation. Therefore, we conducted the present meta-analysis of randomized controlled trials (RCT) to assess the efficacy of needle knife therapy plus SH injection compared with needle knife alone on patients with KOA.

## 2 Methods

### 2.1 Study registration

The protocol for this review has been registered in the International Prospective Register of Systematic Reviews (registration number: CRD42020169602) on April 28, 2020. Available online: https://www.crd. york.ac.uk/PROSPERO/#myprospero. This protocol is reported in accordance with the Preferred Reporting Items for Systematic Review and Meta-Analysis Protocols 2015 statement^[23]^ and the Cochrane Handbook for Systematic Reviews of Interventions.^[24]^

### 2.2 Study selection

#### 2.2.1 Inclusion criteria for studies

1. Patients: The patients who met the diagnostic criteria of KOA.
2. Type of studies: The trials will be RCTs that compared needle knife plus sodium hyaluronate injection with needle knife alone. There are no restrictions on languages.
3. Type of participants: There is no limit to the sex, age, and source of cases.
4. Intervention measures: The treatment group will be treated with needle knife plus SH injection, while the control group with needle knife alone.

#### 2.2.2 Exclusion criteria for studies

1. The studies are not RCT.
2. There is no clear diagnostic or efficacy criteria.
3. There is no definite criterion of curative effect.
4. The control group underwent other different therapies.
5. Lots of RCT papers with the similar research by the same author (we choose one representative article).
6. Animal experiments.

#### 2.2.3 Type of outcome measurements

1. The primary outcomes are the Lysholm knee score (LKSS) and visual analogue scale (VAS).
2. The secondary outcome is clinical effective rate (ER).
  A. Significant improvement: The clinical symptoms of the patients are obviously relieved. There is a little pain occasionally when bearing weight as well as the flexion and extension of the knees are not limited.
  B. Improvement: Although the original symptoms were relieved, the pain and limb function were improved, but not obviously.
  C. No improvement: No improvement in symptoms and physical activity.
  D. Aggravation: The joint symptoms and limb movement were not improvement but aggravate.

### 2.3 Search strategy

The electronic search will be carried out from the following database: PubMed, Web of Science, Cochrane Central Register of Controlled Trials, Embase, ClinicalTrials.gov, The Cochrane Library, Wanfang data, Chinese National Knowledge Infrastructure, and VIP Database, from their inception to 2020. The dates, types, and statuses of the publications will not be limited. The search strategy include the following terms: Osteoarthritis, Knee; Knee Osteoarthritides; Osteoarthritis of Knee; Osteoarthritides of Knee; Knee of Osteoarthritides; Knee of Osteoarthritis; Knees, Osteoarthritides of; Knees, Osteoarthritis of; KOA. Sodium hyaluronate; Hyaluronic Acid; sodium; hyaluronate; Amvisc; Healon. Needle knife; Acupotomy; Acupotomies; Acupuncture; Acupuncture Treatments; Pharmacoacupuncture; Pharmacoacupuncture Treatments; acupoint; needling; electroacupuncture; hand acupuncture. (Randomized controlled trial (RCT); randomly; random; randomized;

### 2.4 Selection of studies

According to the criteria of the selection, we will choose 2 separate reviewers independently to read the title and abstract, screen the documents and extract the data. The third reviewer will discuss and decide whether there is disagreement. First, we will import the retrieved questions into Noteexpress software for duplicate check, and then read the questions and abstracts. We will download the full text according to the incline criteria, select the text for the second time, and then make sure that if the texts are value to be included. The study selection process will be reported according to the Preferred Reporting Items for Systematic Reviews and Meta-Analyses (PRISMA) guidelines.^[25]^

### 2.5 Data extraction

**Figure 1:**
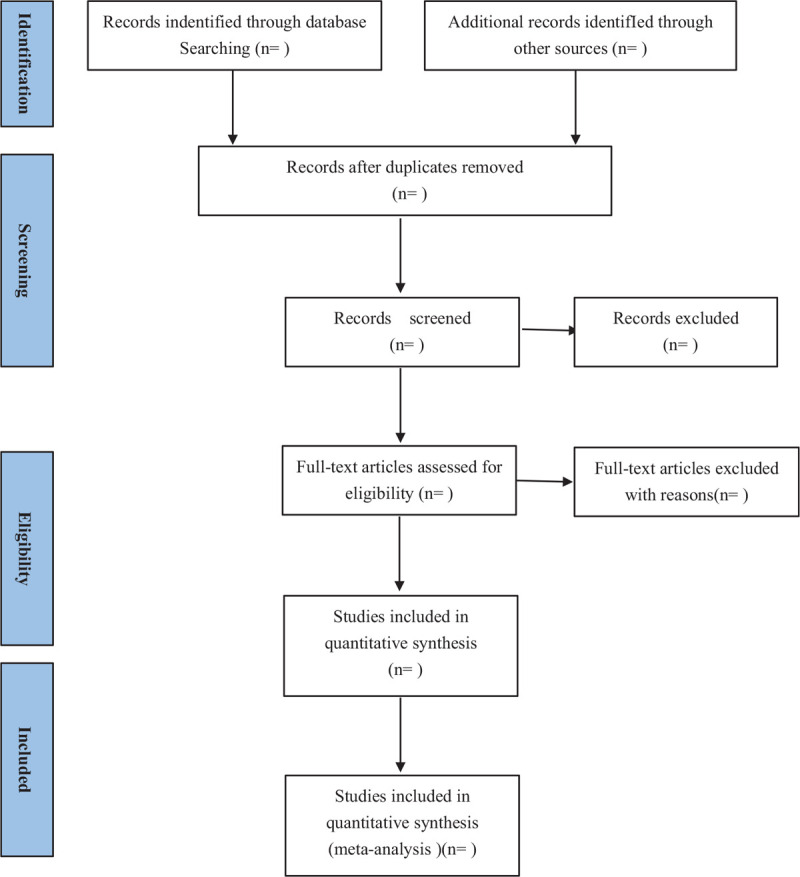
Flowchart of the study selection.

The details of these data will be extracted by 2 separate reviewers from the identified studies based on a unified form according to the predetermined criteria. We will record the first author's name, time of publication, sample size, number of randomly assigned cases, intervention, control procedures, details of treatment, outcome measures, adverse events, sex and age. Disagreements will be resolved through discussion. A third reviewer will be consulted if discrepancies persisted and then decide whether the data should be included. If the data in the report are not clear, contact the authors to obtain the information by email but if the author cannot be contacted, the data will be discussed by the reviewers and then get the reasonable results. The flow chart of all research selection processes is shown in Figure 1.

### 2.6 Assessment of risk of bias

Risk of bias will be assessed independently by 2 reviewers using the Cochrane risk of bias tool according to the Cochrane Handbook of Systematic Reviews of Interventions for the following criteria: random sequence generation, allocation concealment, blinding of participants and personnel, blinding of outcome assessment, incomplete outcome data, selective reporting, and other bias. The judging criteria will be categorized as “low risk of bias,” “high risk of bias,” or “unclear risk of bias.” The discrepancies will be resolved by negotiating and consulting a third reviewer.

### 2.7 Measures of treatment effects

The continuous data and dichotomous data will be included in the outcomes of interest. We use weight mean difference (MD) with 95% to express the continuous data, the pooled relative risk (RR), and 95% confidence interval (CI) for the clinical efficacy rate (ER). Although different methods for the measure of outcomes will be used in different trails, the standardized mean difference (SMD) will be chosen if they have the same outcomes.

### 2.8 Assessment of heterogeneity

The chi-square test and the inconsistency index statistic (*I*^2^) will be used to assess the potential heterogeneity. The fixed-effects model will be applied to express the data without obvious heterogeneity (*P* > .1 and *I*^2^ < 50%). For *P* > .1 and *I*^2^ > 50%, the trails will be considered to be heterogeneous and a random-effects model will be used.

### 2.9 Assessment of reporting bias

We will make funnel plots to assess the publication bias if more than 10 original studies are included. When reporting bias is implied by asymmetry of funnel plot, we will try to fifind out the reason. If the funnel plots indicate publication bias, we will analyze the possible reason and give reasonable explanation.

### 2.10 Data synthesis

We will use RevMan5.3 software provided by Cochrane collaboration for the Meta analysis. First, the heterogeneity will be carried out between experimental groups. When there is no heterogeneity between the experimental groups, the fixed effect model meta-analysis will be selected, otherwise the source of heterogeneity will be found as much as possible. If there is no clinical or methodological heterogeneity, the random effect model will be used.

### 2.11 Sensitivity analysis

In order to test the stability of the meta-analysis results and find out the factors affecting the results, we will conduct the sensitivity analysis by excluding:

1. The researches with high risks of bias.
2. The outliers that are numerically distant from the rest of the data.

### 2.12 Subgroup analysis

When the study is heterogeneous, the subgroup analysis will be performed to find out reasons of heterogeneity. We will divide each study into different groups according to the characteristics of the study and compare the differences of combining effects.

### 2.13 Grading the quality of evidence

The GRADE (Grading of Recommendations Assessment, Development and Evaluation) will be used to evaluate the quality of the studies. The evidence quality evaluation of key outcome indicators can be classified into 4 levels: high (++++), moderate (+++), low (++), and very low (+) as by the GRADE Working Group. Two independent reviewers make the quality of evidence from 5 aspects: research limitations, inconsistencies, indirectness, imprecision, and publication bias. The third reviewer will make a decision after a cross-check if there is dispute.

### 2.14 Ethics and dissemination

Animals and individuals are not contained in the study, so the ethical approval will not be required. The results of the study will be published in conferences or peer-reviewed journals at once as soon as they are finished.

## 3 Discussion

KOA is the osteoarthrosis with a high incidence rate in middle age. The treatment strategy of KOA is to relieve symptoms and delay the process of the joint degeneration. Compared with corticosteroids injection, sodium hyaluronate injection is better in alleviating symptoms and dalaying onset but with the shortcoming of long medication time. It is confirmed that needle knife did great efficiency on KOA. With more and more people suffering from KOA, patients are trying different methods to treat it. But it is hard to find a therapy with both curative effect and few side effects. As clinical routine, needle knife and SH injection are used frequently on patient. However, only a small number of studies have compared the effect of needle knife plus SH with needle knife therapy alone in the treatment of KOA. The protocol of this systematic review and meta-analysis study aims to assess the efficacy, safety of needle knife plus SH in the treatment of KOA. We have tried our best to search the main data base and found that no relevant systematic review and meta-analysis about this topic has been reported in the last 5 years. So in order to provide evidence for clinical application, we are going to perform this systematic review and meta-analysis.

## Author contributions

**Conceptualization:** Kunming Xie

**Data curation:** Xuyue Pan, Fasen Huang, Jingwei Tao.

**Methodology:** Zhaojun Chen.

**Resources:** Huan Liang, Shulong Wang, Zhen Lei.

**Software:** Junde Wu, Zhanhua Ma, Hanjie Zhu

**Supervision:** Yufeng Ma, Yinze Qi, Xinyu Li
